# The Chances of Subsequent Cancer Detection in Patients with a PSA > 20 ng/ml and an Initial Negative Biopsy

**DOI:** 10.1100/tsw.2009.47

**Published:** 2009-05-20

**Authors:** Nadeem Shaida, Catherine M. Jones, Navin Ravindranath, Peter R. Malone

**Affiliations:** Harold Hopkins Department of Urology, Royal Berkshire Foundation NHS Trust, Reading, RG1 5AN, United Kingdom

**Keywords:** prostate specific antigen, prostate cancer, transrectal biopsy, PSA density

## Abstract

Transrectal ultrasound (TRUS)–guided prostate biopsy is known to carry a significant false-negative rate, leading some patients to have multiple biopsies. We investigated cancer detection rates in patients with a PSA >20 ng/ml and a negative initial biopsy. We reviewed our database of 2396 TRUS-guided biopsies done between 1997 and 2002 in order to give a follow-up of at least 6 years. PSA, PSA density (PSAD), PSA velocity (PSAV), prostate volume, and DRE findings were analysed in relation to cancer status. Of the patients, 388 (16%) had a PSA >20 ng/ml, including 99 (26%) with benign biopsies. Of those, 67 were rebiopsied, including 19 (28%) with cancer on the first rebiopsy and four (6%) on further biopsies. PSAD, DRE, and volume significantly differed between rebiopsied patients with and without cancer (*p* <0.05). Patients who present with a PSA >20 ng/ml and have an initial negative biopsy have a high chance of malignancy being detected on a second biopsy. However, if a second biopsy is also negative, then the chances of subsequent biopsies showing signs of cancer are very low if the DRE is normal and particularly if the PSAD is >0.35 ng/ml/cm.

## INTRODUCTION

Prostate cancer is now the commonest male cancer in the U.K. In the main, the diagnosis is dependent on histological examination of prostate tissue which is frequently obtained by transrectal ultrasound (TRUS)–guided needle biopsy. The indications for biopsy are generally accepted as either an elevated prostate-specific antigen (PSA) level or an abnormal-feeling prostate on digital rectal examination (DRE). Despite improvements in technique aimed at increasing cancer detection rates, such as the use of age-specific PSA ranges and extended-core biopsy protocols, the technique still carries a significant false-negative rate[1,2]. This poses the clinical dilemma: What to do with patients who have an initial negative prostate biopsy, yet whose PSA remains elevated or rises further? There is evidence to suggest that in these patients, a second biopsy yields detection rates of up to 25%[3,4]. Given that the chances of detecting prostate cancer rise as the PSA rises, one might expect a higher pickup rate on subsequent biopsies in patients with a high initial PSA level. If the second biopsy is negative, then one might intuitively expect there would be a significant proportion of patients who would be diagnosed on a third or subsequent biopsy. Unfortunately, there is little evidence available to guide clinicians as to the best course of action in such situations.

In order to shed light on this subject, we investigated the subsequent cancer detection rates in patients with an initial PSA > 20 ng/ml and a negative initial biopsy to help determine the likelihood of cancer detection on subsequent biopsies in this group of patients.

## METHODS

A retrospective analysis of the TRUS and prostate biopsy database at the Royal Berkshire Hospital, Reading, U.K. was conducted for all biopsies performed between 1997 and 2002 to ensure a follow-up of at least 6 years. Patients were referred on the basis of either an elevated age-specific PSA[5] or an abnormal DRE. At that time, all biopsies were performed using a standard sextant technique with a spring-loaded 18-guage needle and biopsy gun (Inter-V Pro-Mag™ Ultra, Gainesville, Florida). Subsequent biopsies over the last 5 years have used the extended 10-core technique[2]. The patients age, PSA, DRE finding, and prostate volume as calculated by a modified prolate spheroid formula[6] were recorded.

Patients with a PSA of at least 20 ng/ml and a negative TRUS biopsy were identified for further analysis. The patient notes were retrieved and evaluated for number of biopsies, course of management, serial serum PSA measurements, and final clinical outcome. Further histological results were obtained from the histology department to ensure all available histology results were included for analysis.

Patient age, serum PSA on index biopsy, prostate volume, and DRE findings were correlated with diagnosis of prostate biopsy after the index-negative biopsy. The serial PSA results were used to calculate PSA velocity (PSAV), PSA density (PSAD), and PSAD velocity (PSADV). These variables were correlated with incidence of prostate cancer on subsequent biopsies. PSAV and PSADV required at least two values taken over a period of at least 1 year for calculation.

For each variable, the result at the index biopsy (the first biopsy at which the serum PSA was at least 20 ng/ml) was used for analysis. Patients with repeated benign findings were subgrouped into the benign group, and patients with subsequent diagnosis of prostate cancer comprised the malignant subgroup. The Chi squared test was used to compare age, DRE finding, prostate volume, serum PSA, PSAD, PSAV, and PSADV between the benign and malignant subgroups. Pearsons correlation analysis was performed against cancer status for each variable with mean comparison $p$ value under 0.10. The presence of prostatic intraepithelial neoplasia (PIN), prostatic inflammation, and prostatitis was also correlated with cancer status. Linear regression analysis was performed with all relevant variables in order to determine the presence of any significant prognostic predictors of cancer status.

PSAV and PSAD were evaluated as diagnostic markers using receiver operating characteristic (ROC) curve analysis. The most appropriate thresholds for prognostic sensitivity and specificity were selected based on the resulting ROC curve. Area under the ROC curve (AUC) was calculated to compare overall diagnostic accuracy between the two markers. The negative predictive value (NPV) of PSAD at the time of the index biopsy was evaluated for all patients with repeated biopsies and the subgroup of patients with at least three histological biopsies.

Statistical analysis was considered significant at a $p$ value of 0.05. All analyses were performed using SPSS version 11.0 (SPSS Inc., Chicago).

## RESULTS

Over the period of the study, 2396 TRUS biopsies were performed and recorded into the database. Of these, 388 patients (16%) had a PSA > 20 ng/ml and of these 388, 99 patients had a benign result on the index biopsy. These 99 patients form the dataset for this analysis. Table 1 shows the histological diagnoses for these patients.

**TABLE 1**
**Benign Histological Results for the 99 Patients Included in the Analysis**

| Histological Diagnosis | No. of Results |
|---|---|
| Normal/BPH | 69 |
| Acute inflammation | 18 |
| Chronic inflammation | 7 |
| PIN | 4 |
| Atypical hyperplasia | 1 |

Thirty-two patients did not undergo another TRUS biopsy. Of these, 15 had a repeat PSA after the first biopsy which had normalized; 17 did not undergo a repeat biopsy for medical or social contraindications (including terminal malignancy, warfarinisation, and moving out of the catchment area).

Of the 67 patients who were rebiopsied, 19 (28%) had prostate cancer diagnosed on the first rebiopsy (Table 2). These patients were treated accordingly and then excluded from further analysis. The remaining 48 patients were managed with rebiopsy in 28 cases and with PSA surveillance in 20. Of the 28 patients who underwent a third TRUS biopsy, only two were diagnosed with prostate cancer (Table 2). The remaining 26 patients were treated conservatively in 17 cases and with rebiopsy in nine. These nine patients with a fourth biopsy result showed only one case of malignancy. Only one of four patients with a fifth biopsy had a malignant result, and the single patient with six biopsies had benign results for each one.

**TABLE 2**
**Patients with PSA at Least 20 ng/ml Biopsied from 1997 to 2002**

| | No. of Patients | Benign Results (%) | Malignant Results (%) |
|---|---|---|---|
| Index biopsy | 388 | 99 (25.5) | 289 (74.5) |
| Second biopsy | 67 | 48 (71.6) | 19 (28.4) |
| Third biopsy | 28 | 26 (92.9) | 2 (7.1) |
| Fourth biopsy | 9 | 8 (88.9) | 1 (11.1) |
| Fifth biopsy | 4 | 3 (75) | 1 (25) |
| Sixth biopsy | 1 | 1 (100) | 0 (0) |

The patients who were treated conservatively after each benign biopsy began PSA monitoring, with a repeat biopsy performed if the PSA appeared to be rising steadily. The PSA values were used to calculate PSAV in 59 cases.

Table 3 shows the results for variable differences between benign vs. malignant subgroups. Multivariate analysis of age, PSA, PSAV, PSAD, PSADV, DRE, and prostatic volume against final cancer status showed that only prostate volume was a significant predictor in the multivariate analysis ($p = 0.04$, b = –0.40). In a separate multivariate analysis, findings of PIN, prostatitis, or inflammation were not significant predictors of future cancer diagnosis ($p = 0.28$, $p = 0.09$, $p = 0.06$, respectively).

ROC analysis of PSAV as a predictor of prostate cancer produced AUC of 0.73. A PSAV of 0.78 ng/ml/year or more produced a sensitivity of 71% and specificity of 74%. ROC analysis of index PSAD produced AUC of 0.76. A PSAD of 0.35 ng/ml/$cm^3$ or more on the first biopsy was associated with sensitivity of 87% and specificity of 53%.

**TABLE 3**
**Subgroup Analysis (Comparison of Means and Correlation Analysis)**

| Variable | No. of Patients | Benign Mean (SD) | Malignant Mean (SD) | *p* Value | Pearsons Correlation* |
|---|---|---|---|---|---|
| Index PSA | 67 | 32.4 (15.8) | 39.8 (27.6) | 0.25 | — |
| Age | 67 | 70.5 (6.15) | 71.4 (6.00) | 0.55 | — |
| Abnormal DRE | 67 | 20/44 | 18/23 | **0.007** | **0.01** |
| Prostate volume | 67 | 106.9 (60.3) | 64.3 (22.7) | **<0.0001** | **0.002**** |
| PSAV | 59 | –1.09 (5.91) | 2.52 (12.9) | 0.24 | — |
| PSAD | 67 | 0.51 (0.43) | 0.98 (1.24) | **0.026** | **0.027** |
| PSADV | 43 | –0.0005 (0.001) | –0.0002 (0.0008) | 0.33 | — |

* Correlation with subsequent diagnosis of prostate cancer.

** Negative correlation with subsequent diagnosis of prostate cancer.

A PSAD result <0.35 ng/ml/cm$^3$ at the first biopsy was associated with a NPV of 88% in all 67 patients with repeated biopsies. In the 28 patients who had three or more biopsies, its diagnostic accuracy (both positive and negative predictive value) was 100%. All four patients with subsequent cancer on the third or subsequent biopsy had a PSAD > 0.35 ng/ml/cm$^3$, whilst the remaining 24 patients had a PSAD under that value.

A normal DRE finding at the time of the first biopsy was associated with a NPV of 83% in patients with two or more biopsies, and 91% in patients with three or more biopsies.

## DISCUSSION

Prostate biopsy remains the standard diagnostic test for prostate cancer; nevertheless, it is not without its flaws, in particular the fact that it carries a significant false-negative rate[1,2] due to the fact that prostate cancer is often multifocal and the prostate volume actually sampled by the biopsy is relatively small. In addition, the procedure has associated complications, such as infection, bleeding, and urinary retention[7], making multiple biopsies undesirable for the patient. The conundrum for the clinician, therefore, is to decide how many biopsies to perform before being satisfied that there is no evidence of prostate cancer. The situation is not made easier when one considers other factors that may be influencing PSA values, such as the use of 5-α reductase inhibitors which are accepted to reduce the true PSA reading by as much as 50%, or the effect of chronic inflammation. Furthermore, the discovery of PIN or cellular atypia on an initial or subsequent biopsy would clearly have an impact on the decision to proceed to a further biopsy. In our study, each patient was reviewed in clinic by an experienced clinician to evaluate these factors before a decision was made to proceed to susbsequent biopsy.

Data from other studies suggest that cancer detection rates on biopsies subsequent to a second biopsy are low. Djavan et al.[8] reported that for patients who presented with a PSA between 4 and 10 ng/ml, detection rates were 5% at third biopsy and 4% at fourth biopsy, and Keetch et al.[9] reported figures of 8 and 7%, respectively, for patients presenting with a PSA > 4 ng/ml. However, these studies have not focused on that subgroup of patients with much higher levels of PSA (>20 ng/ml) where the index of suspicion would be greater. It is this cohort where the clinical dilemma is felt most acutely.

A further confounding factor in recent times has been the adoption of extended-core or saturation-sampling techniques, use of which Tan et al.[10] recently reported a 21% detection rate on third biopsy. Interestingly, in this series, the cancers detected on repeat biopsy were smaller, but of higher grade than those detected on initial biopsy, a finding that has not been universally replicated[9,11,12]. Other authors[13,14] advocate the use of saturation biopsy only on repeat biopsy in cases where there is high clinical suspicion despite a negative initial biopsy and it may be the case, that in the end, this will prove to be the most pragmatic approach.

Our results showed cancer detection rates of 26, 28, 7, and 11% at first, second, third, and fourth biopsies, respectively. Although there was a rate of 25% at fifth biopsy, there were only four patients in this group. These figures, perhaps surprisingly, show that even in "high-risk" patients, the vast majority of tumours are picked up on first or second biopsy. With regards to other parameters, there were significant positive associations between abnormal DRE and PSAD and cancer status, and a significant negative relationship (as expected) between prostate volume and cancer status. Using a PSAD cutoff of 0.35 $ng/ml/cm^3$ yielded a NPV of 88% and a normal DRE yielded a NPV of 83% in all patients who had undergone two or more biopsies.

## CONCLUSION

We conclude, therefore, that patients who present with a PSA > 20 ng/ml and have an initial negative biopsy have a high chance of malignancy being detected on a second biopsy. However, if they have a second negative biopsy, the chances of subsequent biopsy showing signs of cancer are very low if the DRE is normal and particularly if the PSAD is >0.35 $ng/ml/cm^3$.
